# Kaplan–Meier survival analysis and Cox regression analyses regarding right ventricular septal pacing: Data from Japanese pacemaker cohort

**DOI:** 10.1016/j.dib.2016.07.058

**Published:** 2016-08-03

**Authors:** Akira Mizukami, Yuya Matsue, Yoshihisa Naruse, Shinya Kowase, Kenji Kurosaki, Makoto Suzuki, Akihiko Matsumura, Akihiko Nogami, Kazutaka Aonuma, Yuji Hashimoto

**Affiliations:** a Department of Cardiology, Kameda Medical Center, 929 Higashi-cho, Kamogawa, Chiba 296-8602, Japan; b Cardiovascular Division, Faculty of Medicine, University of Tsukuba, 1-1-1Tennodai, Tsukuba, Ibaraki 305-8577, Japan; c Department of Cardiovascular Medicine, Yokohama Rosai Hospital, 3211 Kozukue-cho, Kohoku-Ku, Yokohama, Kanagawa 222-0036, Japan

**Keywords:** RV septal pacing, RV non-apical pacing, Prognosis, Heart failure, Mortality, Data

## Abstract

The presented data were obtained from 982 consecutive patients receiving their first pacemaker implantation with right ventricular (RV) lead placement between January 2008 and December 2013 at two centers in Japan. Patients were divided into RV apical and septal pacing groups. Data of Kaplan–Meier survival analysis and Cox regression analysis are presented. Refer to the research article “Implications of right ventricular septal pacing for medium-term prognosis: propensity-matched analysis” (Mizukami et al., in press) [1] for further interpretation and discussion.

**Specifications Table**

**Table:** 

Subject area	*Clinical cardiology, cardiovascular electrophysiology*
More specific subject area	*Cardiac pacing*
Type of data	*Table, figures*
How data was acquired	*Retrospective review of medical records*
Data format	*Analyzed*
Experimental factors	*Japanese pacemaker patients*
Experimental features	*Patients were divided into septal and apical pacing groups, and the prognosis were compared*
Data source location	*Kamogawa and Yokohama city, Japan*
Data accessibility	*Data is with this article*

**Value of the data**

• *The prognostic implication of RV septal pacing remains unclear.*
• *Provided data provides insight on this topic from a large cohort of patients in real world situation.*
• *These data may serve as a benchmark for further data and studies regarding prognosis of RV septal pacing.*

## 1 Data

**Fig. 1:**
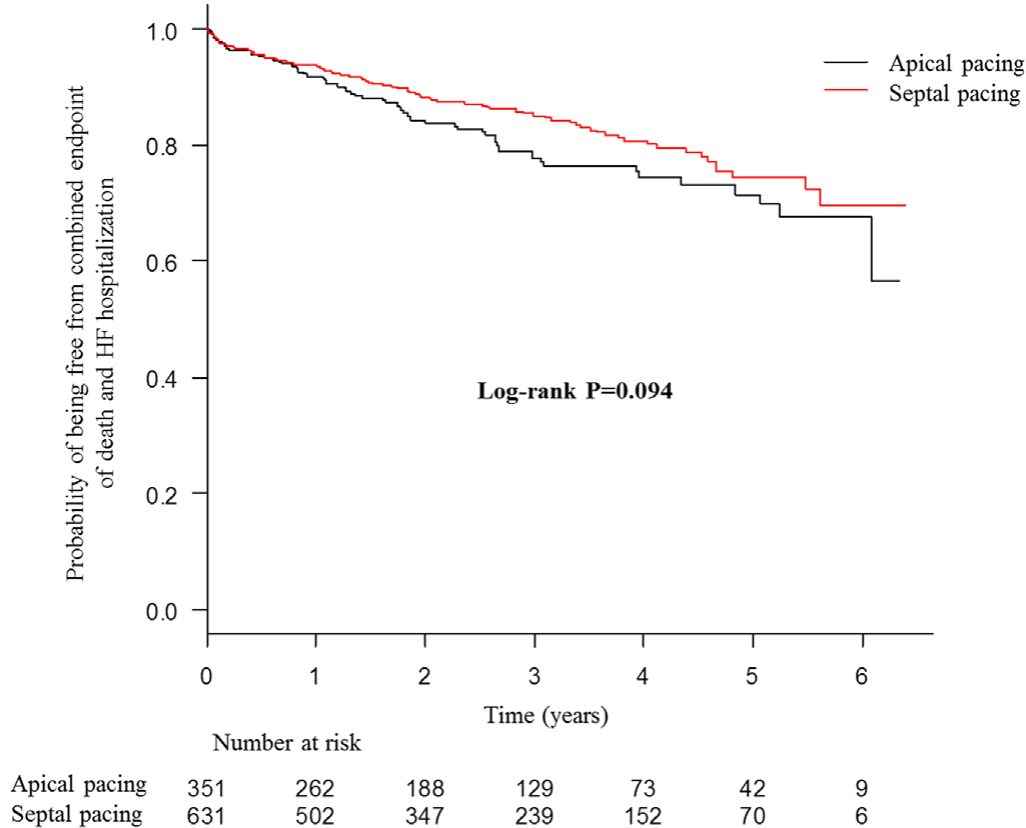
Kaplan–Meier curves for combined primary endpoint of all-cause death and heart failure hospitalization of whole cohort. No significant difference was observed between the two pacing sites.

**Fig. 2:**
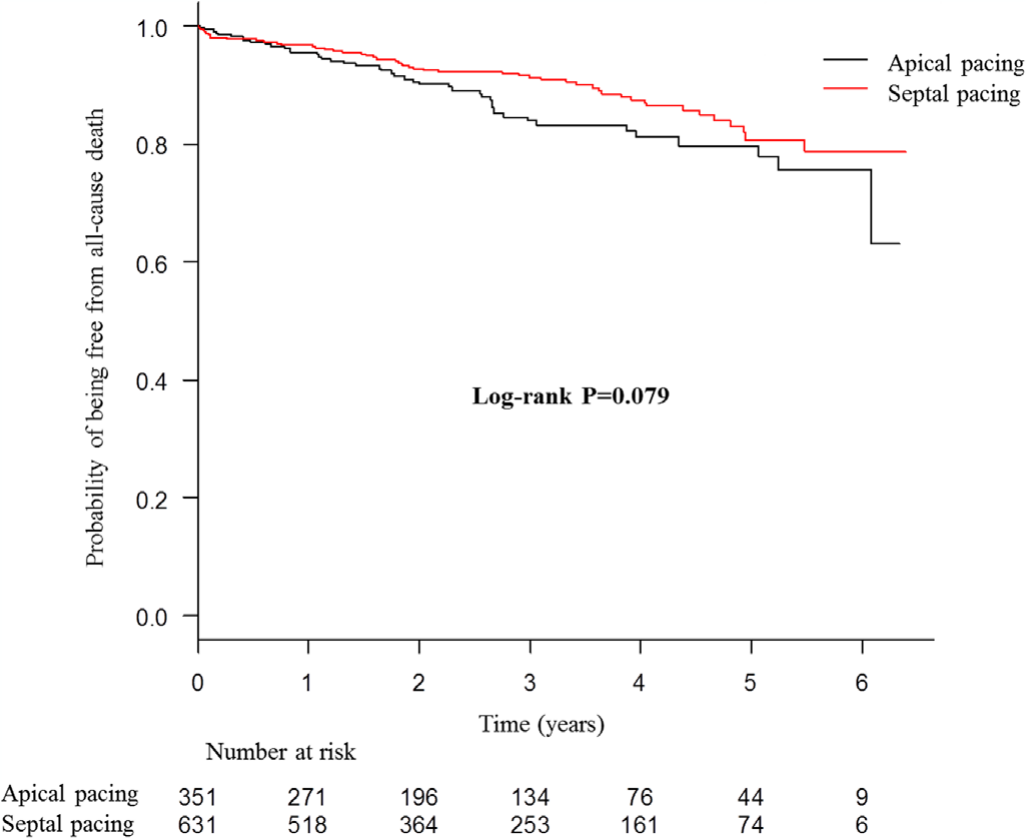
Kaplan–Meier curves for all-cause death of whole cohort. No significant difference was observed between the two pacing sites.

**Fig. 3:**
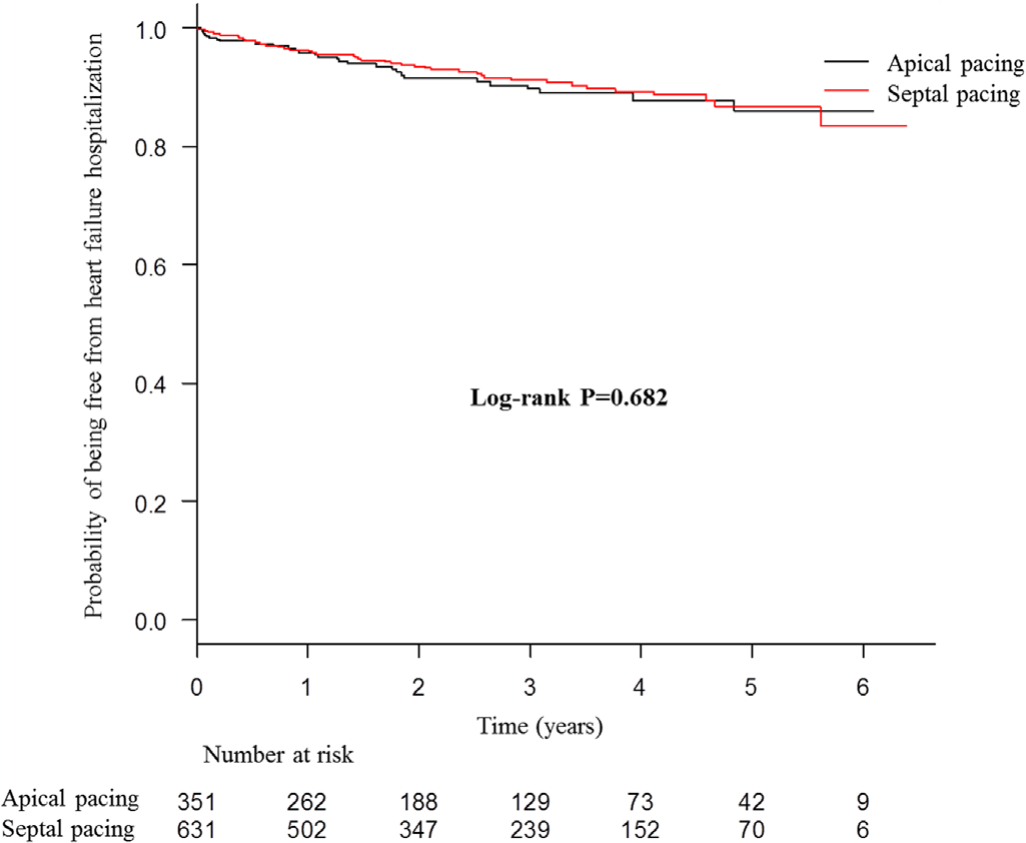
Kaplan–Meier curves for heart failure hospitalization of whole cohort. No significant difference was observed between the two pacing sites.

The presented data were obtained from 982 consecutive patients receiving their first pacemaker implantation with right ventricular (RV) lead placement between January 2008 and December 2013 at two centers in Japan. Patients were divided into RV apical and septal pacing groups. Data of Kaplan–Meier survival analysis for primary combined endpoint of all-cause death and hospitalization due to heart failure (Fig. 1), and secondary endpoints of all-cause death (Fig. 2), and hospitalization due to heart failure (Fig. 3), as well as Cox regression analysis for the primary endpoint (Table 1) are presented. Superiority of septal pacing was not observed in Kaplan–Meier survival analysis and Cox regression analysis for the primary and secondary endpoints. Refer to [1] for further interpretation and discussion.

## 2 Experimental design, materials and methods

We retrospectively included 982 consecutive patients receiving their first pacemaker implantation with RV lead placement between January 2008 and December 2013 at two centers in Japan (Kameda Medical Center and Yokohama Rosai Hospital; 51.4% male, age 76.1±10.6 years, 64.3% septal pacing). The indications for pacemaker implantation were decided according to the guidelines of the Japanese Circulation Society [2]. The target site of RV lead placement was decided by the caring physician on the bases of patient background and operator preference. The location of the RV lead and was assessed at the time of implantation by right anterior oblique and left anterior oblique fluoroscopic projections, as well as paced QRS morphology during implantation using the methods reported previously [3], and was followed-up by biplane chest radiography and 12-lead ECG after implantation. RV outflow tract pacing was included in the RV septal pacing group.

The primary endpoint was a combination of all-cause death and hospitalization due to heart failure. The secondary endpoints included the individual components of the primary endpoint.

Data at the time of implantation procedure were collected, including age, sex, diagnosis for implantation (AV block, sick sinus syndrome [SSS], or others), past history (hypertension, hyperlipidemia, diabetes mellitus, heart failure, atrial fibrillation, and ischemic heart disease), medications (beta-blockers, angiotensin converting enzyme inhibitors/angiotensin receptor blockers, and calcium channel blockers), ECG parameters (QRS interval, presence of complete left bundle branch block [CLBBB]), laboratory parameters (hemoglobin, estimated glomerular filtration rate [eGFR], and B-type natriuretic peptide [BNP]), and left ventricular ejection fraction (LVEF) on transthoracic echocardiography. The diagnosis of AV block included any degree of AV block with indication for pacemaker implantation. Hypertension, hyperlipidemia, and diabetes mellitus were scored based on the previous diagnosis and initiation for therapy. Heart failure, atrial fibrillation, and ischemic heart disease were scored based on previous history. The Modification of Diet in Renal Disease (MDRD) study equation with Japanese coefficient was used to calculate eGFR. This new Japanese equation is currently recommended by the Japanese Society of Nephrology for accuracy in the Japanese population [4].

Data regarding outcome were obtained by a single investigator who was unaware of the patients׳ information, including RV pacing site.

“Time 0” for survival analyses was the date of pacemaker implantation. Comparison of the probability of freedom from the prognostic binary endpoints between groups was performed by Kaplan–Meier survival analysis with estimation of the hazard ratio from a Cox regression model. Univariate and multivariate Cox regression analyses were also performed to determine the prognostic implications of each variable, including RV septal pacing, on the endpoints. Variables with *P*<0.1 on univariate analysis were entered into multivariate analysis. Logarithmic transformations of the variables without a normal distribution were used for Cox regression analysis. The estimates of the parameters were given with their 95% confidence intervals. All *P*-values reported are 2-sided, and *P*<0.05 was considered statistically significant. All statistical analyses were performed with R (The R Foundation for Statistical Computing, Vienna, Austria, version 3.1.1).

## Figures and Tables

**Table 1:** Univariate and multivariate Cox proportional regression analyses of the combined primary endpoint of all-cause death and heart failure hospitalization in the whole cohort.

	**Univariate analysis**			**Multivariate analysis**		
	HR	95%CI	*P-value*	HR	95%CI	*P-value*
RV septal pacing	0.76	0.56–1.05	0.095	1.35	0.90–2.04	0.149
Age	1.06	1.04–1.07	<0.001	1.02	1.00–1.05	0.060
History of diabetes mellitus	1.94	1.40–2.69	<0.001	1.27	0.82–1.99	0.287
History of atrial fibrillation	1.25	1.06–1.49	0.010	1.62	1.08–2.43	0.021
History of heart failure	5.10	3.72–6.98	<0.001	2.61	1.73–3.95	<0.001
History of ischemic heart disease	2.69	1.93–3.75	<0.001	1.43	0.91–2.27	0.124
β-Blocker	1.77	1.26–2.49	0.001	0.89	0.57–1.39	0.600
Log QRS duration	2.71	1.36–5.41	0.005	1.95	0.76–4.96	0.164
Hemoglobin	0.73	0.67–0.79	<0.001	0.96	0.85–1.07	0.431
Log estimated GFR	0.19	0.13–0.28	<0.001	0.34	0.16–0.71	0.004
Log BNP	1.88	1.60–2.21	<0.001	1.31	1.05–1.64	0.019
Left ventricular EF	0.95	0.94–0.97	<0.001	0.98	0.96–0.99	0.003

RV, right ventricular; GFR, glomerular filtration rate; EF, ejection fraction; HR, hazard ratio; CI, confidence interval.
