# Supplementary material for: Kaplan–Meier survival analysis and Cox regression analyses regarding right ventricular septal pacing: Data from Japanese pacemaker cohort
Source: Data Brief. 2016 Aug 3;8:1303–7. doi: 10.1016/j.dib.2016.07.058 (PMC4990661; doi:10.1016/j.dib.2016.07.058)
Supplement: Supplementary file 1 — Supplementary material [file mmc1.pdf]

Elsevier Editorial System(tm) for Data in Brief

Manuscript Draft

Manuscript Number: DIB-D-16-00526R2

Title: Kaplan-Meier survival analysis and Cox regression analyses regarding right ventricular septal pacing: data from Japanese pacemaker cohort

Article Type: Data Article

Keywords:

Corresponding Author: Dr. Akira Mizukami,

Corresponding Author's Institution:

First Author: Akira Mizukami

Order of Authors: Akira Mizukami

Abstract: The presented data were obtained from 982 consecutive patients receiving their first pacemaker implantation with right ventricular (RV) lead placement between January 2008 and December 2013 at two centers in Japan. Patients were divided into RV apical and septal pacing groups. Data of Kaplan-Meier survival analysis and Cox regression analysis are presented. Refer to the research article "Implications of right ventricular septal pacing for medium-term prognosis: propensity-matched analysis"[1] for further interpretation and discussion

Detailed Response to Reviewers

Reviewer #1: Minor revision required before the article can be accepted.

Please update the title: remove the word 'effects' and ensure the word 'data' or 'dataset' appears in the title.

Also, please provide a clean version when revising, ensuring that track changes are not visible.

Response: We thank the reviewer for the valuable comments. We have updated the title accordingly to “Kaplan–Meier survival analysis and Cox regression analyses regarding right ventricular septal pacing: data from Japanese pacemaker cohort”. We apologize for the visible track changes in the previous revision. We have provided the clean version.

*Manuscript
Click here to view linked References

Data article

Title: ***Kaplan–Meier survival analysis and Cox regression analyses regarding right ventricular septal pacing: data from Japanese pacemaker cohort***

Authors:

Akira Mizukami, MD[1]; Yuya Matsue, MD[1]; Yoshihisa Naruse, MD[2]; Shinya Kowase, MD[3]; Kenji Kurosaki, MD[3]; Makoto Suzuki, MD[1]; Akihiko Matsumura, MD[1]; Akihiko Nogami, MD[2]; Kazutaka Aonuma, MD[2]; Yuji Hashimoto, MD[1]

Affiliations:

1) Department of Cardiology, Kameda Medical Center

929 Higashi-cho, Kamogawa, Chiba 296-8602, Japan

2) Cardiovascular Division, Faculty of Medicine, University of Tsukuba

1-1-1 Tennodai, Tsukuba, Ibaraki 305-8577, Japan

3) Department of Cardiovascular Medicine, Yokohama Rosai Hospital

3211 Kozukue-cho, Kohoku-Ku, Yokohama, Kanagawa 222-0036, Japan

Contact email: akmizukami@gmail.com

Abstract

The presented data were obtained from 982 consecutive patients receiving their first pacemaker implantation with right ventricular (RV) lead placement between January 2008 and December 2013 at two centers in Japan. Patients were divided into RV apical and septal pacing groups. Data of Kaplan–Meier survival analysis and Cox regression analysis are presented. Refer to the research article "Implications of right ventricular septal pacing for medium-term prognosis: propensity-matched analysis"[1] for further interpretation and discussion.

Specifications Table

Subject area	*Clinical cardiology, cardiovascular electrophysiology*
More specific subject area	*Cardiac pacing*
Type of data	*Table, figures*
How data was acquired	*Retrospective review of medical records*
Data format	*Analyzed*
Experimental factors	*Japanese pacemaker patients*
Experimental features	*Patients were divided into septal and apical pacing groups, and the prognosis were compared*

Data source location	*Kamogawa and Yokohama city, Japan*
Data accessibility	*Data is with this article*

Value of the data

- *The prognostic implication of RV septal pacing remains unclear.*
- *Provided data provides insight on this topic from a large cohort of patients in real world situation.*
- *These data may serve as a benchmark for further data and studies regarding prognosis of RV septal pacing.*

Data

The presented data were obtained from 982 consecutive patients receiving their first pacemaker implantation with right ventricular (RV) lead placement between January 2008 and December 2013 at two centers in Japan. Patients were divided into RV apical and septal pacing groups. Data of Kaplan–Meier survival analysis for primary combined endpoint of all-cause death and hospitalization due to heart failure (Figure 1), and secondary endpoints of all-cause death (Figure 2), and hospitalization due to heart failure (Figure 3), as well as Cox regression analysis for the primary endpoint (Table 1) are presented. Superiority of septal pacing was not observed in Kaplan–Meier survival analysis and Cox regression analysis for the primary and secondary endpoints. Refer to [1] for further interpretation and discussion.

Experimental Design, Materials and Methods

We retrospectively included 982 consecutive patients receiving their first pacemaker implantation with RV lead placement between January 2008 and December 2013 at two centers in Japan (Kameda Medical Center and Yokohama Rosai Hospital; 51.4% male, age 76.1 ± 10.6 years, 64.3% septal pacing). The indications for pacemaker implantation were decided according to the guidelines of the Japanese Circulation Society.[2] The target site of RV lead placement was decided by the caring physician on the bases of patient background and operator preference. The location of the RV lead and was assessed at the time of implantation by right anterior oblique and left anterior oblique fluoroscopic projections, as well as paced QRS morphology during implantation using the methods reported previously,[3] and was followed-up by biplane chest radiography and 12-lead ECG after implantation. RV outflow tract pacing was included in the RV septal pacing group.

The primary endpoint was a combination of all-cause death and hospitalization due to heart failure. The secondary endpoints included the individual components of the primary endpoint.
Data at the time of implantation procedure were collected, including age, sex, diagnosis for implantation (AV block, sick sinus syndrome [SSS], or others), past history (hypertension, hyperlipidemia, diabetes mellitus, heart failure, atrial fibrillation, and ischemic heart disease), medications (beta-blockers, angiotensin converting enzyme inhibitors/angiotensin receptor blockers, and calcium channel blockers), ECG parameters (QRS interval, presence of complete left bundle branch block [CLBBB]), laboratory parameters (hemoglobin, estimated glomerular filtration rate [eGFR], and B-type natriuretic peptide [BNP]), and left ventricular ejection fraction (LVEF) on transthoracic echocardiography. The diagnosis of AV block included any degree of AV block with indication for pacemaker implantation. Hypertension, hyperlipidemia, and diabetes mellitus were scored based on the previous diagnosis and initiation for therapy. Heart failure, atrial fibrillation,

and ischemic heart disease were scored based on previous history. The Modification of Diet in Renal Disease (MDRD) study equation with Japanese coefficient was used to calculate eGFR. This new Japanese equation is currently recommended by the Japanese Society of Nephrology for accuracy in the Japanese population.[4]

Data regarding outcome were obtained by a single investigator who was unaware of the patients' information, including RV pacing site.
"Time 0" for survival analyses was the date of pacemaker implantation. Comparison of the probability of freedom from the prognostic binary endpoints between groups was performed by Kaplan–Meier survival analysis with estimation of the hazard ratio from a Cox regression model. Univariate and multivariate Cox regression analyses were also performed to determine the prognostic implications of each variable, including RV septal pacing, on the endpoints. Variables with $P < 0.1$ on univariate analysis were entered into multivariate analysis. Logarithmic transformations of the variables without a normal distribution were used for Cox regression analysis. The estimates of the parameters were given with their 95% confidence intervals. All P-values reported are 2-sided, and $P < 0.05$ was considered statistically significant. All statistical analyses were performed with R (The R Foundation for Statistical Computing, Vienna, Austria, version 3.1.1).]

Acknowledgements
None

References
[1] Mizukami A, Matsue Y, Naruse Y, Kowase S, Kurosaki K, Suzuki M, et al. Implications of right ventricular septal pacing for medium-term prognosis: propensity-matched analysis. Intl J Cardiology. 2016;in press.
[2] Group JCSJW. Guidelines for Non-Pharmacotherapy of Cardiac Arrhythmias (JCS 2011). Circ J. 2013;77:249-74.
[3] Mond HG, Hillock RJ, Stevenson IH, McGavigan AD. The right ventricular outflow tract: the road to septal pacing. Pacing Clin Electrophysiol. 2007;30:482-91.
[4] Matsuo S, Imai E, Horio M, Yasuda Y, Tomita K, Nitta K, et al. Revised equations for estimated GFR from serum creatinine in Japan. Am J Kidney Dis. 2009;53:982-92.

Figure legends:

Figure 1. Kaplan-Meier curves for combined primary endpoint of all-cause death and heart failure hospitalization of whole cohort

No significant difference was observed between the two pacing sites.

Figure 2. Kaplan–Meier curves for all-cause death of whole cohort

No significant difference was observed between the two pacing sites.

Figure 3. Kaplan–Meier curves for heart failure hospitalization of whole cohort

No significant difference was observed between the two pacing sites.

Table 1. Univariate and multivariate Cox proportional regression analyses of the combined primary endpoint of all-cause death and heart failure hospitalization in the whole cohort

	Univariate analysis			Multivariate analysis		
	HR	95%CI	*P-value*	HR	95%CI	*P-value*
RV septal pacing	0.76	0.56 – 1.05	0.095	1.35	0.90 – 2.04	0.149
Age	1.06	1.04 – 1.07	< 0.001	1.02	1.00 – 1.05	0.060
History of diabetes mellitus	1.94	1.40 – 2.69	< 0.001	1.27	0.82 – 1.99	0.287
History of atrial fibrillation	1.25	1.06 – 1.49	0.010	1.62	1.08 – 2.43	0.021
History of heart failure	5.10	3.72 – 6.98	< 0.001	2.61	1.73 – 3.95	< 0.001
History of ischemic heart disease	2.69	1.93 – 3.75	< 0.001	1.43	0.91 – 2.27	0.124
β-Blocker	1.77	1.26 – 2.49	0.001	0.89	0.57 – 1.39	0.600
Log QRS duration	2.71	1.36 – 5.41	0.005	1.95	0.76 – 4.96	0.164
Hemoglobin	0.73	0.67 – 0.79	< 0.001	0.96	0.85 – 1.07	0.431
Log estimated GFR	0.19	0.13 – 0.28	< 0.001	0.34	0.16 – 0.71	0.004
Log BNP	1.88	1.60 – 2.21	< 0.001	1.31	1.05 – 1.64	0.019
Left ventricular EF	0.95	0.94 – 0.97	< 0.001	0.98	0.96 – 0.99	0.003

RV, right ventricular; GFR, glomerular filtration rate; EF, ejection fraction; HR, hazard ratio; CI, confidence interval.

Figure 1.

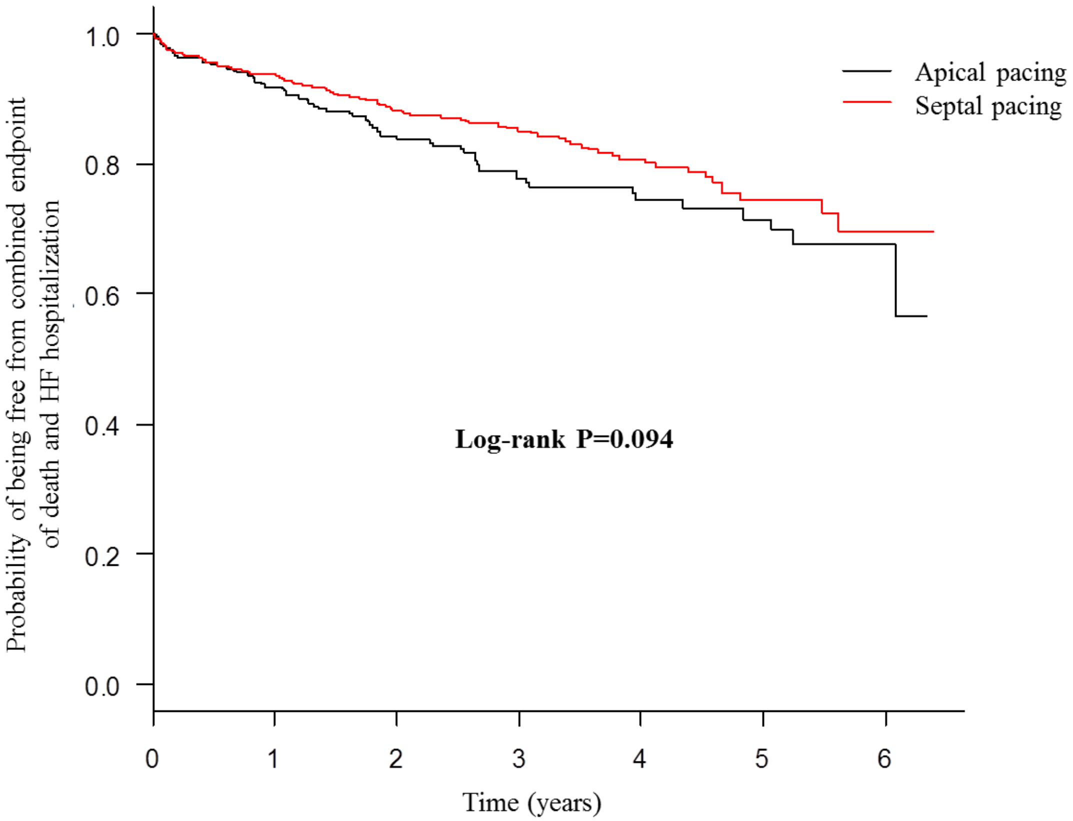

Number at risk

Apical pacing	351	262	188	129	73	42	9
Septal pacing	631	502	347	239	152	70	6

Figure 2.

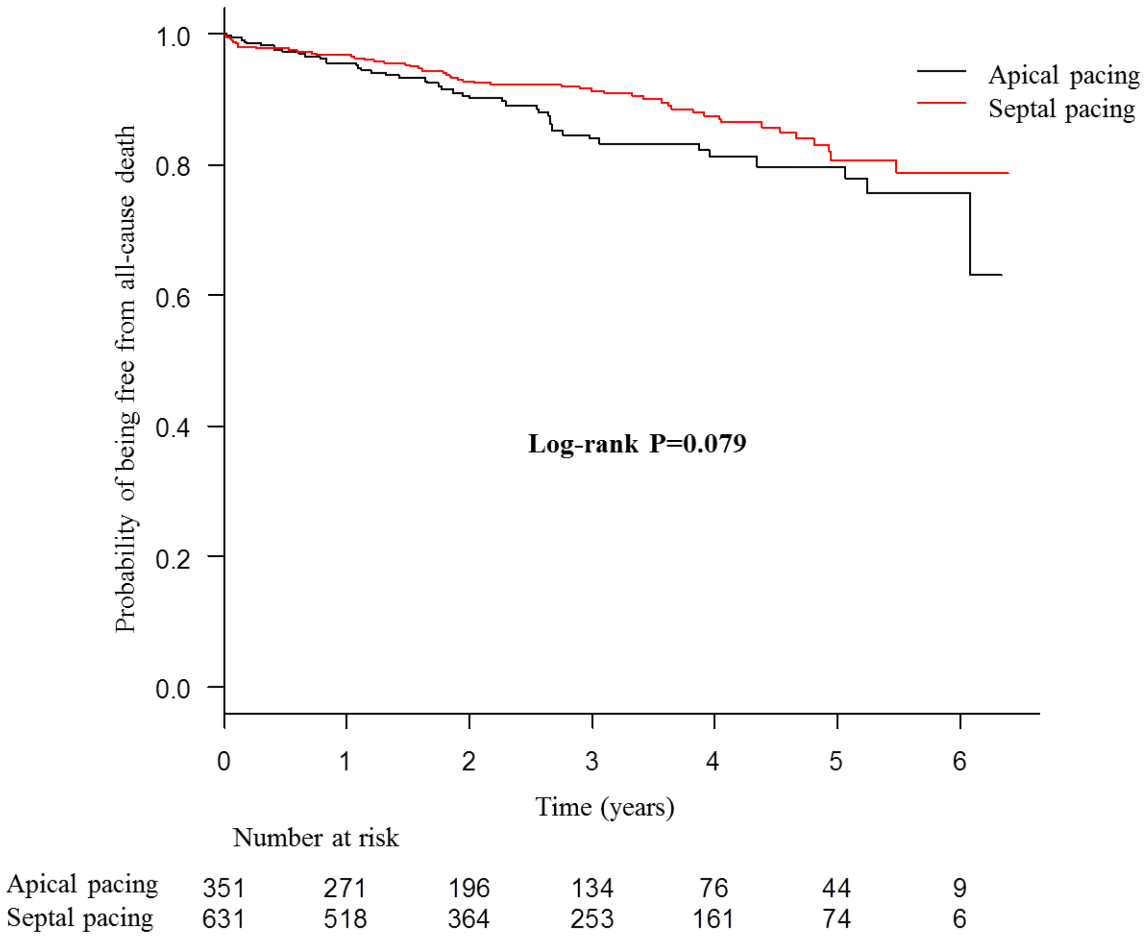

Apical pacing
Septal pacing
Probability of being free from all-cause death
1.0
0.8
0.6
0.4
0.2
0.0
Log-rank P=0.079
0
1
2
3
4
5
6
Time (years)
Number at risk
Apical pacing 351 271 196 134 76 44 9
Septal pacing 631 518 364 253 161 74 6

Figure 3.

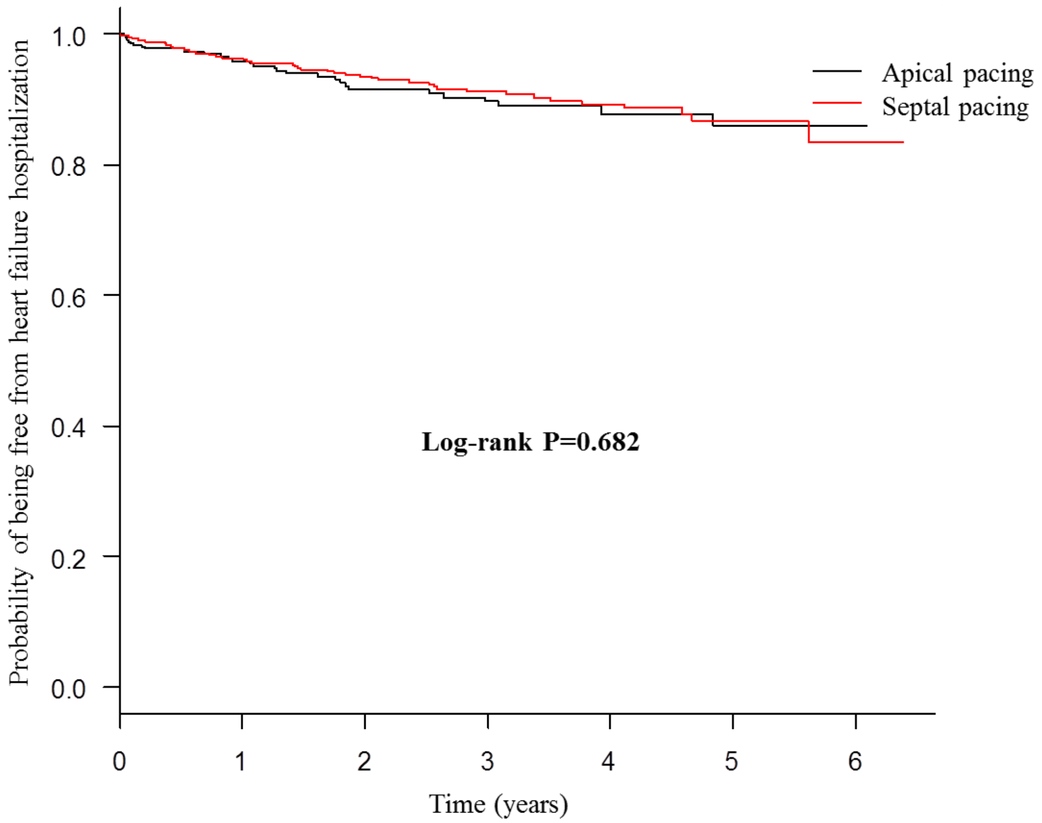

Number at risk

Apical pacing	351	262	188	129	73	42	9
Septal pacing	631	502	347	239	152	70	6

*Conflict of Interest Form
Click here to download Conflict of Interest Form: COI form.pdf
